# Nutrition intervention is beneficial to the quality of life of patients with gastrointestinal cancer undergoing chemotherapy in Vietnam

**DOI:** 10.1002/cam4.3766

**Published:** 2021-02-07

**Authors:** Linh Thuy Nguyen, Anh Kim Dang, Phuong Thi Duong, Hanh Bich Thi Phan, Chinh Tuyet Thi Pham, Anh Tuan Le Nguyen, Huong Thi Le

**Affiliations:** ^1^ Institute for Preventive Medicine and Public Health Hanoi Medical University Hanoi Vietnam; ^2^ Department of Nutrition and Dietetic Hanoi Medical University Hospital Hanoi Vietnam

**Keywords:** digestive cancer, gastric cancer, nutrition, quality of life

## Abstract

**Introduction:**

The best treatment therapy for gastrointestinal cancer patients is assessed by the improvement of health status and quality of life (QoL) after treatments. Malnutrition is related to loss of muscle strengths which leads to lower physical performance and emotional status. Thus, this study aimed to estimate the effects of nutritional interventions on the improvement of QoL among gastrointestinal patients undergoing chemotherapy in Vietnam.

**Methods:**

A quasi‐experiment with intervention and control groups for pre‐ and post‐intervention assessment was carried out at the Department of Oncology and Palliative Care—Hanoi Medical University Hospital from 2016 to 2019. Sixty gastrointestinal cancer patients were recruited in each group. The intervention regimen consisted of nutritional counseling, a specific menu with a recommended amount of energy, protein, and formula milk used within 2 months. Nutritional status and QoL of patients were evaluated using The Scored Patient‐Generated Subjective Global Assessment (PG‐SGA) and The European Organization for Research and Treatment of Cancer (EORTC). The difference in differences (DiD) method was utilized to estimate the outcome between control and intervention groups.

**Results:**

After the intervention, patients of the intervention group had better changes in scores of global health status (Coef =16.68; 95% CI =7.90; 25.46), physical (Coef =14.51; 95% CI =5.34; 23.70), and role functioning (Coef =14.67; 95% CI =1.63; 27.70) compared to the control group. Regarding symptom scales, the level of fatigue, pain, and insomnia symptoms significantly reduced between pre‐ and post‐intervention in the intervention group. In addition, living in urban areas, defined as malnourished and having low prealbumin levels, were positively associated with the lower global health status/QoL score.

**Conclusion:**

Nutritional therapy with high protein was beneficial to the improvement in QoL, physical function and the reduction of negative symptoms among gastrointestinal cancer patients. Early individualized nutritional support in consultation with professional dietitians during chemotherapy plays an integral part in enhancing the QoL and better treatment prognosis.

Clinical trial registration number: NCT04517708.

## Graphical abstract

Nutritional therapy with high‐protein and energy intake is beneficial to the improvement in quality of life (QoL) and physical function as well as the reduction of negative symptoms among gastrointestinal cancer patients. Early individualized nutritional support in consultation with professional dietitians during chemotherapy plays an integral part in enhancing the QoL and better treatment prognosis of the patients.

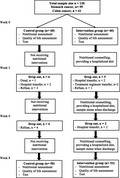

## 1 INTRODUCTION

According to GLOBOCAN 2018, stomach and colon cancer rank the fourth and the fifth common cancer worldwide, respectively.^1^ More importantly, stomach and colon cancer are the second and the fifth leading cause of cancer‐related mortality globally in 2018.^1^ Epidemiological data in Vietnam shows similar trends in which stomach and colon remain the third and the eighth leading cause of malignancy death in both sexes.^2^ Gastrointestinal cancer is often asymptomatic or has nonspecific symptoms at an early stage, contributing to the delayed diagnosis and relatively poor prognosis and mortality.^3^ It is suggested that the best treatment therapy is assessed by the survival rate and quality of life (QoL) of patients after the treatment.^4^ QoL is a term that subjectively evaluates the well‐being of an individual in terms of not only physical, but also psychological aspects.^5^ Therefore, the QoL is emphasized in reflecting the health status of cancer patients, and nutritional factors substantially affect the QoL of cancer patients.^6^

Patients with gastrointestinal cancer are at a higher risk of experiencing weight loss and malnutrition in the early process due to the blockages and interference with the flow of food in the digestive tract by the tumors.^7^ In addition, malnourishment is attributable to several concurrent issues such as loss of appetite, reduced nutrient malabsorption, gastrointestinal symptoms, and anemia.^4^ One of the common nutritional performance statuses that can be seen in cancer patients is cachexia. This is a complex condition consisting of the reducing intake, the increase of energy requirement, and metabolic dysfunction, which may lead to a higher rate of mortality of cancer patients.^8^ Treatment‐induced changes in nutrition and metabolism may trigger alterations in physiological and psychological functions, which can negatively impact patients’ well‐being and result in the reduction of QoL.^9^ Regarding physical function, loss of muscle strengths induced by cachexia may limit patients’ physical performance.^10^ Besides, chemotherapy treatment methods may have adverse effects on the nutritional status of gastrointestinal system cancer patients directly via weight loss due to lack of appetite, changes in sense of taste and smell, side effects of chemotherapy such as nausea, vomiting, and mucositis.^11^ Cancer patients under chemotherapy may face several psychological issues, including stress, anxiety, depression, or physiological side effects such as hair loss, pain, tiredness, nausea, vomiting, or social side effects of role and function loss.^12^ Although the objective of chemotherapy is cancer curation and increase the survival rate, it can mitigate the QoL of patients.^13^

It has been shown that diet improvements play a critical role in maintaining a higher QoL of patients by boosting their emotional status, social structure, and enjoyment.^14^ A systematic review examining the QoL of gastric cancer patients concluded that better nutritional status was associated with a better QoL.^15^ By contrast, an inflammatory response in cancer patients may contribute to weight loss and preferential loss of protein in muscle, which leads to reduced appetite and lower QoL.^16^ In addition, several clinical signs may also trigger difficulties in the daily life of patients, such as fatigue (feeling of tiredness or lack of energy), nausea (sensation of wanting to vomit), dyspnea (shortness of breath), insomnia (have trouble falling and/or staying asleep), or appetite loss.^17^ Previous studies also showed the close relationship between a healthy diet, a change in lifestyle and longer life, increased survival rate and QoL among colon cancer patients.^18^, ^19^, ^20^

In Vietnam, most of the studies focus on determining the nutritional status or QoL of cancer patients.^21^, ^22^ There is a little study that evaluates the relationship of those two issues on cancer patients. The previous study which investigated the relationship between nutritional status and QoL of gastrointestinal patients, concluded that underweight patients or those at high risk of malnourishment had a lower QoL than normal‐weight and well‐nourished patients.^23^ Based on those findings, this study aimed to estimate the effects of nutritional intervention therapies on the improvement of QoL among gastrointestinal cancer patients in Vietnam.

## 2 METHOD

### 2.1 Study setting and participants

This study was designed as a quasi‐experiment with intervention and control groups, in which participants were not randomly assigned to both groups. Pre‐ (T0) and post‐assessment (T1) were also carried out. The study was conducted at the Department of Oncology and Palliative Care—Hanoi Medical University Hospital from 2016 to 2019. The eligibility for selecting participants included (a) aged 18 years old or above; (b) diagnosed with stomach or colon cancer; (c) at the initial of receiving chemotherapy treatment; (d) indicated for oral feeding; (e) not having comorbidities of chronic diseases such as kidney failure, heart failure, liver failure, diabetes; (f) having ability to communicate with data collectors and both attending doctors and participants agreed to involve in the study. We excluded participants (a) being treated by other methods such as radiation, endocrine, immunity, or (b) who underwent terminal palliative care, or (c) who had contraindications to oral feeding/enteral nutrition.

In this study, we utilized sample size calculation for detecting the difference between the means of two samples (intervention and control group). The criteria for evaluating the intervention's effectiveness was the amount of increased weight after the intervention. We used data from a previous study, which showed that the mean of increased weight after the intervention was 1.3 kg, with a standard deviation of 3.6 kg.^24^

$$n_{1}=\;\frac{r+1}{r}\;\frac{{\left(Z_{1‐\frac{\beta }{2}}+\;Z_{1‐\frac{\alpha }{2}}\right)}^{2\;}\sigma^{2}}{d^{2}}$$

For the above sample size formula,^25^ we applied *d* = 1.3 (kg), *σ* = 3.6 (kg), the *β* strength =0.1 and *α* = 0.05, *r* = ratio between the two groups. The sample sizes of the two groups were equal with a ratio of 1:1. As a result, the required sample size was 45 patients for each group. The final sample size with a refusal rate of 20% was 60 patients for each group.

**FIGURE 1:**
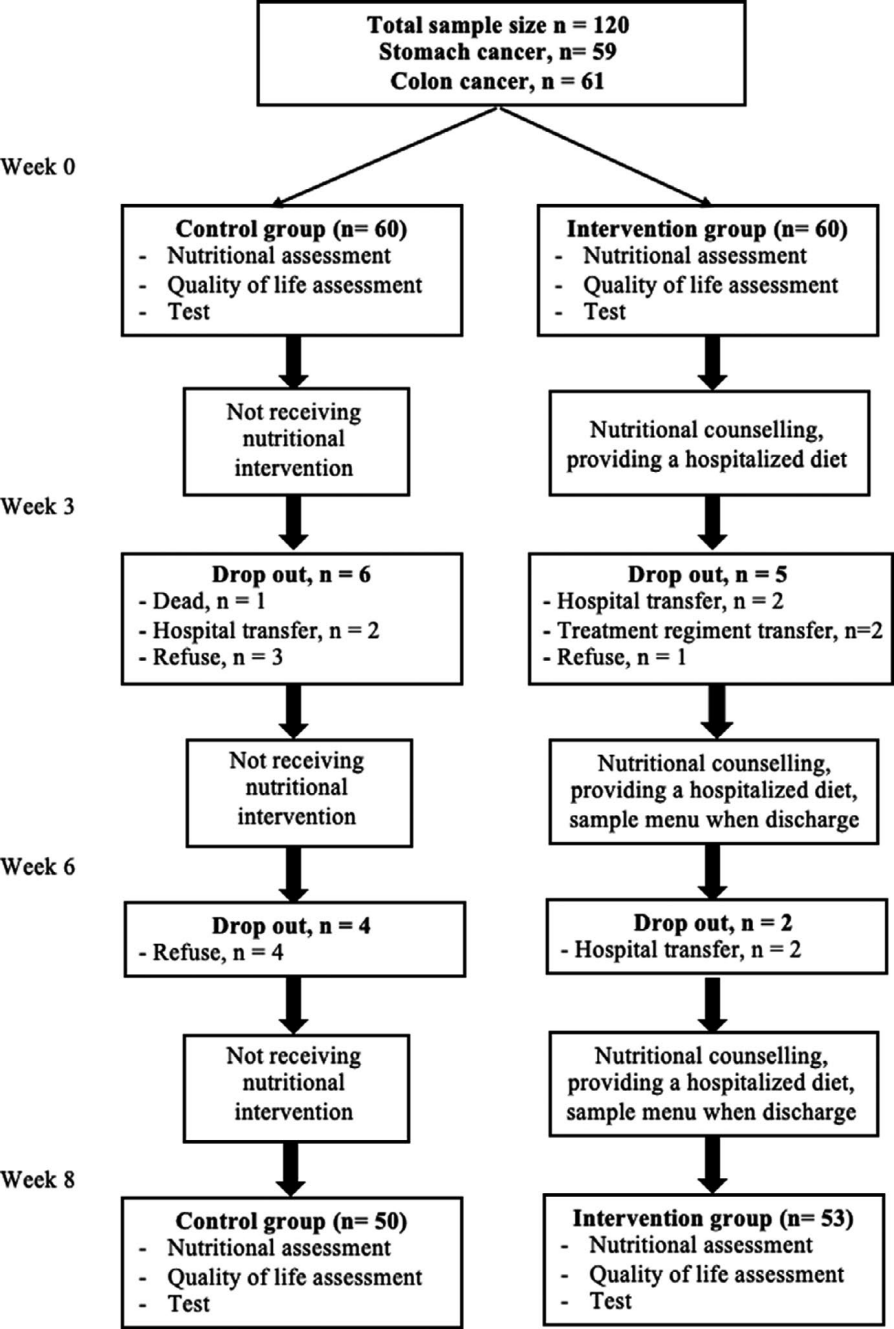
Flow chart of the intervention

A convenience sampling technique was used for recruiting participants. At stage 1, we selected patients of the intervention group who met the inclusion criteria to receive nutritional counseling and diet at the hospital. At stage 2, we chose eligible patients but did not accept the nutritional counseling and diet at the hospital. Participants in both groups were paired together according to the following criteria: (a) Age group: <40 years old, 40–65 years old, and >65 years old; (b) Gender: Male and female; (c) Types of cancer: stomach cancer and colon cancer; (d) Disease stages: stage 1–2 and stage 3–4. The intervention flow chart which included the number of participants in each stage, is presented in Figure 1.

### 2.2 Developing the intervention protocol

*Step 1*. We briefly overviewed the international methods and tools which have been primarily used for screening, assessing nutritional status, and nutritional care processes.

*Step 2*. The intervention protocol was developed based on the documents explored in step 1 and the current practice guidelines of nutritional care for cancer patients at Hanoi Medical University Hospital.

*Step 3*. We consulted more than 30 nutritional experts on developing the intervention protocol and made the revised versions.

*Step 4*. We developed the tools, documents, and materials related to the intervention, including:

- Consultancy leaflets.
- Menus based on energy levels following the demand recommendations by The European Society for Clinical Nutrition and Metabolism (ESPEN).^26^
  - Total energy: 30 kcal/kg of body weight/day.
  - Protein: 1.2–1.6 g/kg of body weight/day.

The advantage of these menus was the higher energy levels, compared to the normal diets with the same weight and volume. Depending on the weight and digestive condition of each patient, we classified the menus into different energy levels, which consisted of 1500–1600 kcal/day, 1700–1800 kcal/day, or 1900–2000 kcal/day (Appendix S1). In particular, high‐energy meals were made from common foods in the form of soup. The nutritional plan of each patient was also devised with protein‐energy‐rich snacks. If the supplements were feasible and sensible, patients consumed two snacks ×200 ml formula milk (Ingredients of formula milk presented in Appendix S2).

- Preparing the menus and high‐energy soup preparations and evaluating these menus based on suitability and acceptance of the patients.
- Guidelines for processing high‐energy soup preparations from common foods (Appendix S3).

*Step 5*. Applying the protocol to intervention study.

### 2.3 Strategies of the nutrition intervention program

Professional dietitians assessed nutritional status within the first 24‐h after the patients admitting to the hospital at two‐time points (baseline—T0 and 2 months after chemotherapy—T1). All participants were advised by the same dietitian using a predetermined standardized procedure to secure the consistency of individualized nutritional intervention. Relevant aspects of nutritional status (anthropometry indicators, clinical characteristics) and QoL were reported by a defined form. In the control group, patients had diets based on their demands. Regarding the intervention group, patients were treated with the intervention regimen, which consisted of:

- Nutritional counseling.
- Each patient was assigned a specific menu prepared by research members during the time of staying at the hospital. Before discharge, patients were instructed on preparing their diets at home with the recommended amount of energy and protein and given formula milk within 2 months.
- To monitor the intervention process, the dieticians continuously evaluated the daily diet of each patient during hospitalization and conducted telecounseling every 2 weeks for weight monitoring and nutritional support advice when discharge. Patients’ nutritional status were reevaluated at every hospital admission for chemotherapy.
- The target of the nutritional intervention was ensuring the ability to meet the recommended energy and protein of patients. The criteria for making adjustments to the intervention was practicable and being implemented by the patient. In addition, an appropriate treatment tailored to the patients’ eating preferences and digestive and absorptive capacity was taken into consideration.
- Two months after the initiation of nutritional therapy, the implementation of diets were evaluated using the same structured questionnaire (T1).

### 2.4 Measurements and instruments

A 20‐min interview‐administered questionnaire was conducted to collect the data. To test the questionnaire, we carried out a pilot study among 20 participants with different socioeconomic backgrounds. Only minor changes in the wording were made based on the feedback of participants. The following information was included in the study:

#### 2.4.1 Socioeconomic characteristics

Participants self‐reported information regarding their age, gender, educational level, employment, living area (rural/urban), types of cancer, and disease duration.

#### 2.4.2 Assessment of nutrition

We evaluated the anthropometry of participants included height and weight, Mid‐Upper Arm Circumference (MUAC), and muscle mass.

1. We measured standing height using a standard height ruler with an accuracy of 0.1 cm. For weight measurement, we used an electronic scale with an accuracy of 0.1 kg, and calibration was done prior to measurement. Both height and weight measurements were done twice, and the average value was recorded.
2. We used a MUAC tape, a soft and non‐stretch tape with an accuracy of 0.1 cm to measure MUAC. The mid‐point between the tip of the shoulder and the tip of the elbow in the left upper arm was determined and marked. Stretching the patient's arm, looping the tape around the marked point with moderate tension, and reading the result to the nearest 0.1 cm.
3. Muscle mass covered the measurement of the smooth and skeletal muscles as well as water in the body. Bone mass is the overall bone mineral density measurement of the body. In this study, to determine the skeletal muscles mass, total body water, and bone mass of participants, bioelectrical impedance analysis (BIA) of the Tania scale (BC 758) was utilized, which is common in body composition estimation and assessment of clinical conditions due to the noninvasiveness, the portability of bioimpedance analysis systems as well as low cost.^27^ Participants were instructed to stand with bare feet on four metal electrodes. A trained researcher monitored these measurements according to the instructions of device manufacturers. The resistance and reactance when an electrical signal passed through the body were estimated by the impedance value (*Z*).^28^ Because of commercial reasons, the majority of manufacturers did not publish the standardized calculations to derive results of body composition,^28^ but the weight as well as height^2^ were incorporated in the formulae.^29^

The level of serum prealbumin was assessed and classified as following (a) 11–15 mg/dl: increased risk of malnutrition; (b) 5–10.9 mg/dl: fair risk of malnutrition; and (c) <5 mg/dl: severe malnutrition risk.^30^

The Scored Patient‐Generated Subjective Global Assessment (PG‐SGA) was used as the preeminent interdisciplinary patient assessment among patients with oncology or other chronic catabolic conditions.^31^ This tool includes (a) four patient‐generated historical components (Weight History, Food Intake, Symptoms, Activities, and Function); (b) the professional part (Diagnosis, Metabolic stress, and Physical Exam); (c) the Global Assessment (A = well‐nourished, B = moderately malnourished, and C = severely malnourished); (d) the total score, and nutritional triage recommendations.

#### 2.4.3 Quality of life

We utilized the European Organization for Research and Treatment of Cancer (EORTC) version 3.0, which is an integrated and modular approach to holistically evaluate the QoL of patients with cancer.^32^ This tool consists of 30 questions and divides into five functional scales (15 questions), nine symptom scales (13 questions), and a global health status/QoL scale (2 questions). Each question was rated from “Not at all” marked as 1‐point to “Very much” marked as 4‐point. Steps of scoring are presented as follows^33^: (a) Calculating the average score—raw score of each item; (b) Estimating the final score by standardizing the raw score using linear transformations. Thus, all dimensions of the tool will range from 0 to 100, in which a higher score presents a better level of functioning/the global health status or a worse level of symptoms.

### 2.5 Data analysis

The data analysis was conducted by stata software version 12 (StataCorp. LP). In this study, we utilized the difference in differences (DiD) method to estimate the outcome between control and intervention groups.^34^, ^35^ This method compared the outcomes in two groups, which were recorded in two time periods. At first, the differences were calculated in the first and second (before and after intervention). Then, the average gain in the control group was deducted from the average increase in the intervention group. The average treatment effect (ATE) measures the difference in mean (average) outcomes between the QoL of the intervention and the control group. The coefficient value (Coef) describes the relationship between a predictor variable and the response, in which the response changes given a one‐unit change in the predictor. The sign of each coefficient reflects the direction of the relationship, including a positive sign (the predictor variable increases, the response variable also increases) or a negative sign (the predictor variable increases, the response variable decreases).

Chi‐squared test or Fisher's exact test was used for determining the differences of categorical variables. The Mann–Whitney *U* test was used to compare the differences between two continuous variables and not normally distributed. McNemar's chi‐squared was a statistical test used on paired nominal data. Another nonparametric test used for matched data was the Wilcoxon Signed‐Rank Test. We utilized generalized linear mixed effects (GLMM) models with Gaussian distribution and log link function to explore the factors associated with patients’ QoL by adjusting for relationships of other variables with the expected outcome.^36^ Statistically significant is considered when *p* < 0.05.

## 3 RESULTS

Table 1 shows the socioeconomic and clinical characteristics of patients. At the beginning, the number of patients in each group was 60. After 8 weeks, 10 patients in the control group and seven patients in the intervention group were dropped out of the study. Approximately two‐thirds of the participants were males (62.1%). About 48.5% of patients had educational levels under high school. Being retired accounted for the highest percentage (38.8%) of occupation, followed by farmers/workers (25.2%). The majority of participants were at stage 3–4 of cancer duration (83.5%). The mean age was 56.5 (SD =10.4).

**TABLE 1:** Socioeconomic and clinical characteristics of participants (*n* = 103)

	Control group (*n* = 50)		Intervention group (*n* = 53)		Total (*n* = 103)		*p*‐value
	*n*	%	*n*	%	*n*	%	
Gender							
Males	31	62.0	33	62.3	64	62.1	0.98^a^
Females	19	38.0	20	37.7	39	37.9	
Educational level							
Under high school	25	50.0	25	47.2	50	48.5	0.60^a^
High school	13	26.0	10	18.9	23	22.3	
Intermediate college	4	8.0	8	15.0	12	11.7	
University/Post graduated	8	16.0	10	18.9	18	17.5	
Occupation							
Office workers	4	8.0	7	13.2	11	10.7	0.60^b^
Farmers/Workers	12	24.0	14	26.4	26	25.2	
Retired	22	44.0	18	34.0	40	38.8	
Doing household	12	24.0	14	26.4	26	23.3	
Living area							
Rural	25	50.0	27	51.0	52	50.5	0.92^b^
Urban	25	50.0	26	49.0	51	49.5	
Type of cancer							
Stomach cancer	22	44.0	29	54.7	51	49.5	0.28^b^
Colon cancer	28	56.0	24	45.3	52	50.5	
Cancer stage							
Stage 1–2	9	18,0	8	15,1	17	16.5	0.69^b^
Stage 3–4	41	82,0	45	84,9	86	83.5	
	Mean	SD	Mean	SD	Mean	SD	*p*‐value
Age	58.2	10.0	54.9	10.6	56.5	10.4	0.11^c^

^a^ Chi‐squared test.

^b^ Fisher's exact test.

^c^ Mann–Whitney test.

The assessment of the nutritional status of participants is described in Table 2. The weight and muscle mass of patients of the intervention group were significantly higher after receiving nutritional therapies, while these results were not statistically significant among the control group. Regarding the PG‐SGA classification, the percentage of well‐nourished participants in both groups at T1 were statistically higher than that at T0.

**TABLE 2:** Nutritional status of participants before and after intervention (*n* = 103)

	Control group (*n* = 50)		Intervention group (*n* = 53)		Total (*n* = 103)		*p*‐value (1–2)	*p*‐value (3–4)
	T0 (1)	T1 (2)	T0 (3)	T1 (4)	T0	T1		
Weight (kg)	50.5 ± 7.6	50.9 ± 7.1	50.2 ± 7.4	51.6 ± 7.8	50.4 ± 7.5	51.3 ± 7.4	0.19^a^	**<0.01** ^a^
Muscle mass (kg)	37.0 ± 5.7	37.6 ± 5.6	36.5 ± 5.8	37.7 ± 6.6	36.8 ± 5.7	37.6 ± 6.1	0.16e^a^	**0.02** ^a^
MUAC (cm)	25.2 ± 3.1	24.6 ± 3.1	25.3 ± 2.5	25.6 ± 2.9	25.3 ± 2.8	25.1 ± 3.0	0.16^a^	0.29^a^
PG‐SGA classification								
PG‐SGA A	9(18.0)	20 (40.0)	12 (22.6)	34 (64.2)	21 (20.4)	54 (52.4)	**<0.01** ^b^	**<0.01** ^b^
PG‐SGA B	30 (60.0)	21 (42.0)	31 (58.5)	14 (26.4)	61 (59.2)	35 (34.0)		
PG‐SGA C	11 (22.0)	9 (18.0)	10 (18.9)	5 (9.4)	21 (20.4)	14 (13.6)		
Prealbumin (mg/dL)	20.1 ± 5.5	18.1 ± 4.5	22.2 ± 6.3	19.4 ± 6.6	21.2 ± 6.0	19.2 ± 6.3	0.34^a^	**0.0** ^a^

^a^ Wilcoxon signed‐rank test.

^b^ McNemar's chi‐squared test.

Bolded *p*‐value describes the statistical significance.

**FIGURE 2:**
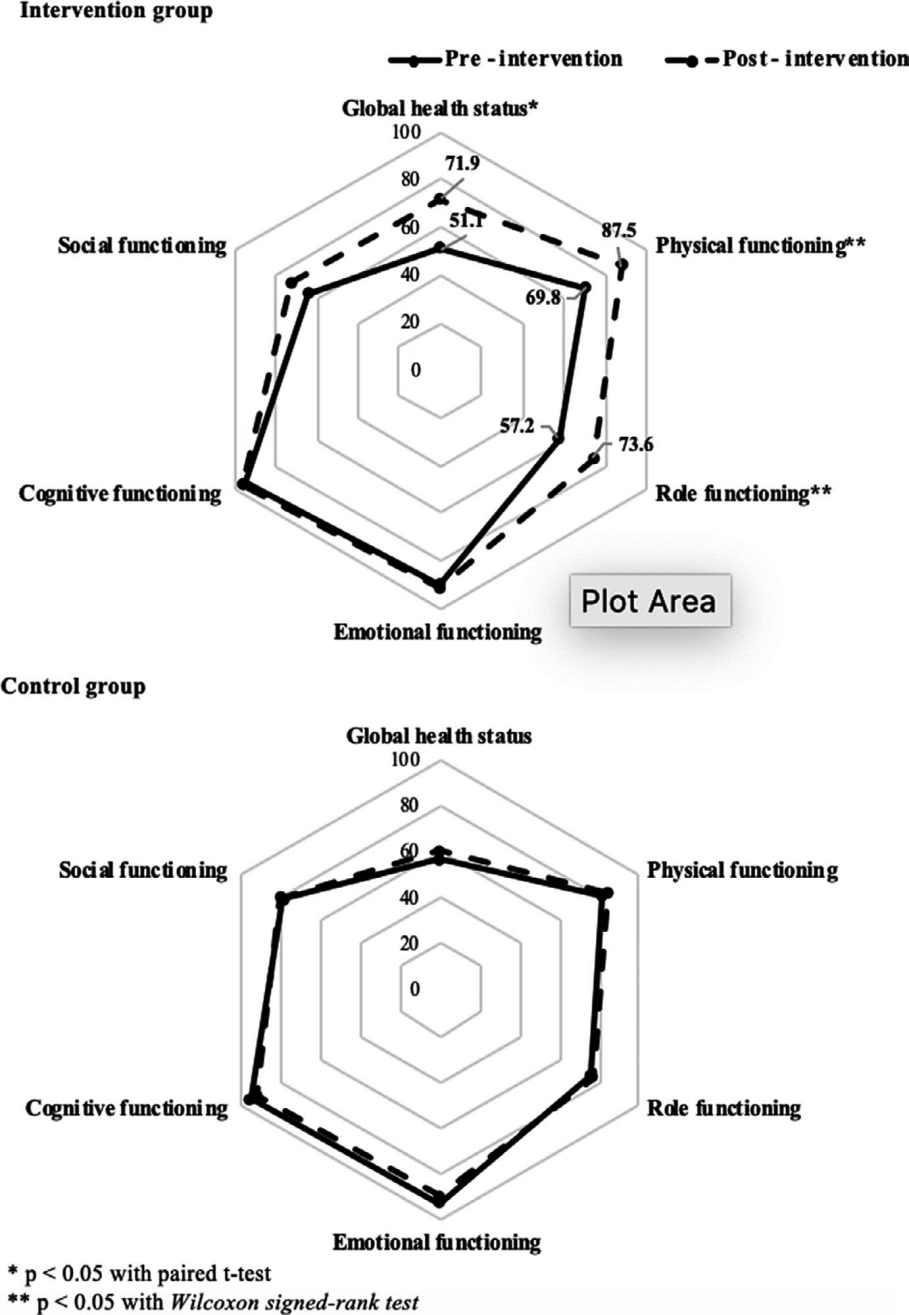
Global health status and Functional scales scores of pre‐ and post‐intervention among Intervention (*n* = 53) and Control group (*n* = 50)

Figure 2 depicts the score of Global health status and Functional scales between pre‐ and post‐intervention among the Intervention and Control group. In the intervention group, the QoL/global health status score of post‐intervention was statistically significantly higher than pre‐intervention (71.9 vs 51.1, respectively). Similarly, the score of physical (87.5 vs 69.8, respectively) and role functioning (73.6 vs 57.2, respectively) after the intervention were also statistically higher than to pre‐intervention. However, these results among the control group were not statistically significant.

**FIGURE 3:**
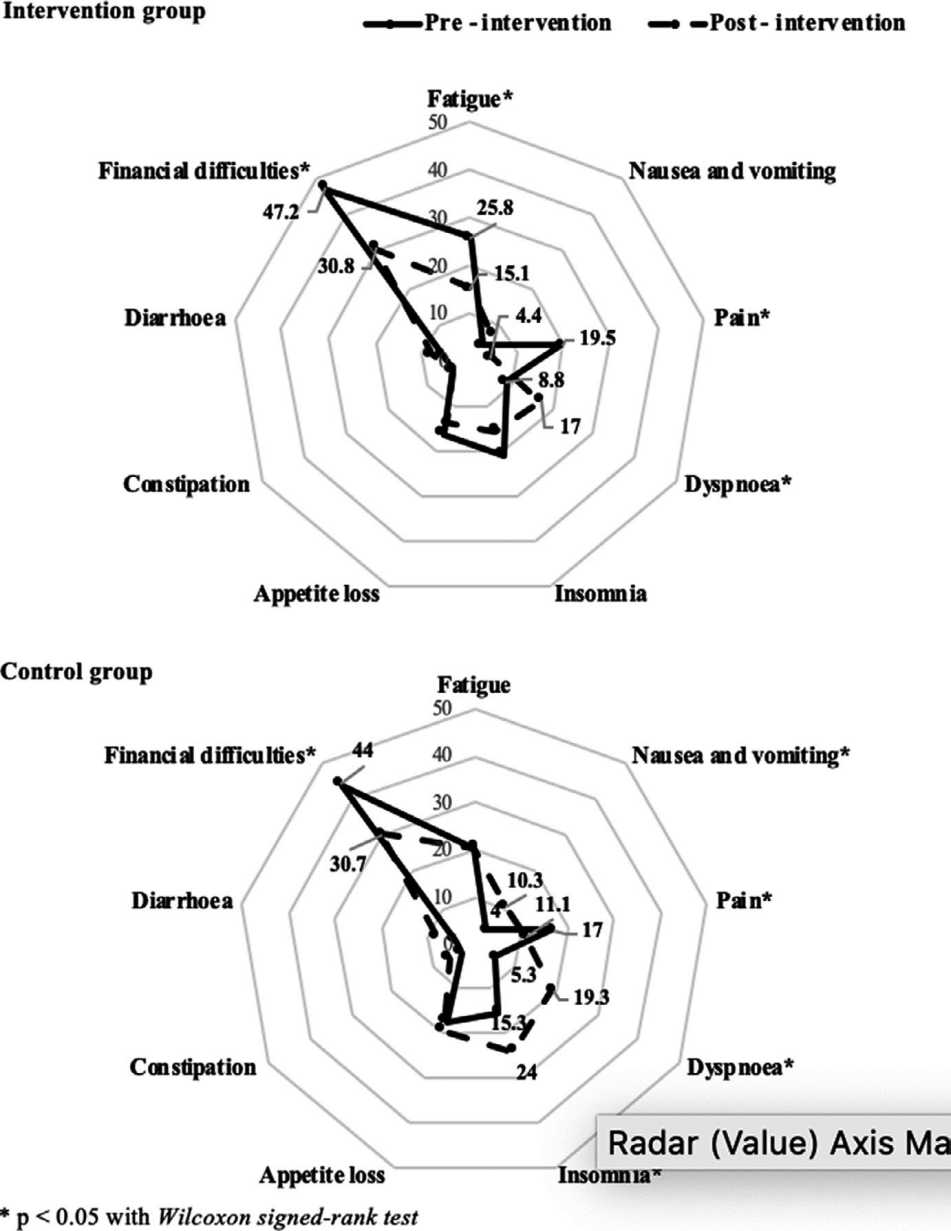
Symptom scales and Financial scores of pre‐ and post‐intervention among Intervention (*n* = 53) and Control group (*n* = 50)

The score of symptom scales and financial of pre‐ and post‐intervention among both groups are presented in Figure 3. Both groups witnessed a decrease in pain scores after the intervention (19.5 vs 4.4, respectively, in intervention group; 17.0 vs 11.1, respectively, in control group). By contrast, the score of dyspnea significantly increased after the intervention in two groups (8.8 vs 17.0, respectively, in intervention group; 5.3 vs 19.3, respectively, in control group). Among the intervention group, patients had fewer fatigue symptoms between pre‐ and post‐intervention (25.8 vs 15.1, respectively). Regarding the control group, the score of nausea/vomiting (4.0 vs 10.3, respectively) and insomnia (15.3 vs 24.0, respectively) also increased, which indicates the worse level of those symptoms.

Tables 3 and 4 show the result of the ATE between pre‐ and post‐intervention using the DiD method. The results indicate that the intervention impacted effectively on the QoL of cancer patients. After the intervention, patients of the intervention group were more likely to have a higher score of global health status (Coef =16.68; 95% CI =7.90; 25.46), physical (Coef =14.51; 95% CI =5.34; 23.70), and role functioning (Coef =14.67; 95% CI =1.63; 27.70) compared to the control group, which presents a higher level of healthy functioning and better QoL. Regarding symptom scales, the level of fatigue (Coef = −10.68; 95% CI = −21.63; −0.28), pain (Coef = −9.03; 95% CI = −17.76; −0.30), insomnia symptoms (Coef = −13.79; 95% CI = −28.07; −0.48) were significantly better between pre‐ and post‐intervention in the intervention group.

**TABLE 3:** Average treatment effect (ATE) between pre‐ and post‐intervention among intervention and control groups using the DiD method regarding global health status and Functional scales

	Global health status		Physical functioning		Role functioning		Emotional functioning		Cognitive functioning		Social functioning	
	Coef	95% CI	Coef	95% CI	Coef	95% CI	Coef	95% CI	Coef	95% CI	Coef	95% CI
Interaction between Intervention and Time	16.68*	7.90; 25.46	14.51*	5.34; 23.70	14.67*	1.63; 27.70	4.1	−4.54; 12.74	3.95	−3.24; 11.14	7.97	−4.83; 20.78

Adjusted for age, gender, occupation, education, living area, type of cancer. There are no difference between intervention and control group among those variables. In which, Intervention (0 = control; 1 = intervention) and Time (0 = pre‐intervention; 1 = post‐intervention).

* *p* < 0.05.

**TABLE 4:** ATE between pre‐ and post‐intervention among intervention and control groups using the DiD method regarding Symptom scales and Financial difficulties

	Fatigue		Nausea and vomiting		Pain		Dyspnea		Insomnia		Appetite loss		Constipation		Diarrhea		Financial difficulties	
	Coef	95% CI	Coef	95% CI	Coef	95% CI	Coef	95% CI	Coef	95% CI	Coef	95% CI	Coef	95% CI	Coef	95% CI	Coef	95% CI
Interaction between Intervention and Time	−10.68*	−21.63 to −0.28	−3.45	−10.65; 3.75	−9.03*	−17.76; −0.30	−5.67	−16.52; 5.19	−13.79*	−28.07; −0.48	−4.56	−18.93; 9.80	−2.02	−9.28; 5.23	−1.44	−10.67; 7.80	−2.69	−15.65; 10.26

Adjusted for age, gender, occupation, education, living area, type of cancer. There are no difference between intervention and control group among those variables. In which, Intervention (0 = control; 1 = intervention) and Time (0 = pre‐intervention; 1 = post‐intervention).

* *p* < 0.05.

Factors associated with Global health status are shown in Table 5. Living in urban areas was associated with having a lower score of Global health status (Coef = −0.09; 95% CI = −0.18; −0.01) compared to those living in rural areas. Moreover, participants who were defined as malnutrition and who had a low level of prealbumin had a worse QoL (Coef = −0.19; 95% CI = −0.28; −0.11 and Coef = −0.12; 95% CI = −0.22; −0.02).

**TABLE 5:** Factors that associated with score of Global health status

	Score of global health status/QoL	
	Coef	95% CI
Gender (male vs female)	0.03	−0.09; 0.14
Age	0.00	−0.01; 0.00
Educational level (vs under high school)		
High school	0.04	−0.07; 0.15
Intermediate college	−0.11	−0.27; 0.04
University/Post graduated	0.09	−0.07; 0.24
Occupation (vs office workers)		
Farmers/Workers	0.00	−0.20; 0.19
Retired	0.15	−0.04; 0.33
Doing household	−0.22	−0.65; 0.20
Living area (Urban vs Rural)	−0.09*	−0.18; −0.01
Type of cancer (Colon cancer vs Stomach cancer)	0.03	−0.06; 0.11
PG‐SGA classification (Malnutrition vs normal)	−0.19*	−0.28; −0.11
Weight	0.01	0.00; 0.02
Muscle mass	−0.01	−0.02; 0.00
MUAC	−0.01	−0.13; 0.11
Prealbumin classification (Low vs normal)	−0.12*	−0.22; −0.02

* *p* < 0.05.

## 4 DISCUSSION

To our knowledge, there is a scarcity of studies in Vietnam, which investigates the effects of nutritional intervention on the QoL of patients with gastrointestinal cancer. Findings from this study provide valuable evidence for clinicians, public health practitioners, and policymakers to conduct early individualized nutritional counseling that enhances not only patients’ nutritional parameters, but also their QoL. We found that nutritional therapy significantly improved the QoL of the intervention group, compared to the control group regarding global health status, physical functioning, and role functioning. By contrast, the mean score of symptoms were significantly higher in the control group than the intervention group toward fatigue, pain, and insomnia. In addition, living in urban areas, defined as malnourished and having low prealbumin levels, were positively associated with the lower score of global health status/QoL.

Our study found that nutritional diet intervention statistically significantly benefited some QoL aspects, compared to those who had usual diets. This result supports the findings of research which defined the positive association between improvement of nutrition status and a higher QoL of cachexia cancer patients receiving chemotherapy (consuming at least one can per day providing 1298 kJ, 16 g protein, and 1.1 g eicosapentaenoic acid).^37^ The finding is also consistent with previous studies of Isenring et al on gastrointestinal cancer patients^38^, ^39^ and Moses et al^40^ in demonstrating the effectiveness of nutritional therapy regarding the increase of QoL among cancer patients. A systematic review and meta‐analysis of 1414 participants from 13 studies presented that oral dietary interventions were related to the significant increase of “emotional functioning,” “global QoL” function scales, and the decrease of “dyspnea” and “loss of appetite” symptom of malnourished patients with cancer.^17^ In contrast, a study by Alexandra et al carrying out nutritional therapies on patients suffered from different types of cancers revealed no benefits of interventions on patients’ QoL, despite a reported higher consumption of dietary intake.^41^ In this study, patients might experience the pressure of fulfilling the goals of nutritional therapy, which could trigger additional emotional stress or discomfort and lead to a decrease of QoL.^41^ Besides, the author noticed that the results should be interpreted with caution due to the small sample size, and other covariables influenced the findings.^41^ It should be noticed that both the control and the intervention groups were better nourished at stage T1. After chemotherapy, most of the participants were taken care of by family members and medical staff. Patients and family members themselves also tried to obtain the appropriate diet for best nourishing through many different information sources. During chemical treatment, most of the current chemical regimens cause side effects on the gastrointestinal tract such as fatigue, anorexia, nausea, vomiting, and digestive disorders. Therefore, the nutritional status may worsen without timely and active intervention, resulting in negative effects on QoL. According to the results, after the intervention, the intervention group had better nutritional status than the control group in terms of weight, muscle mass, and PG‐SGA classification.

In our study, the physical function and role function scores of the intervention group after nutritional therapy were significantly higher than that of the usual care group. This finding is consistent with a previous study, which pointed out the increase of physical function score in nutritional intervention groups was due to the increase in muscle strength of gastrointestinal cancer patients.^39^ We also found that being malnourished was related to a lower score of global health status/QoL. Patients with cancer may experience cachexia, a complex condition consisting of the reduction of intake, the increase of energy requirement, and metabolic dysfunction.^8^ Cachexia is attributed to the alterations in protein and lipid metabolism and the imbalance between the production and degradation processes of muscle proteins.^42^ Cancer patients are more likely to suffer from sarcopenia due to the accelerated proteolysis, lipolysis, and reduced muscle protein synthesis, the primary mechanism for muscle loss.^43^ Sarcopenia is positively associated with low muscle strength and/or impaired physical performance and role function, poor prognosis of the treatment tolerance.^44^ This situation occurs more seriously among gastrointestinal cancer patients because the gastrointestinal symptoms (anorexia, nausea, vomiting, malabsorption, and pain) may present long before the diagnosis of malignancy, which leads to the early cachexia.^45^ Diets of patients in our study were fortified by high‐protein intake (1.2–1.6 g/kg of body weight/day), which placed a significant emphasis on maintenance or growth of muscle mass.^26^ Therefore, patients of the intervention group might reduce the risk of physical impairment, and experience a better performance status as well as role function in their daily life.

Regarding symptom scale, participants in the nutrition support group also reported a decrease in the post‐intervention score of fatigue, pain, and insomnia, compared to the control group. Our results are in accordance with previous research, which revealed that nutritional therapy intervention could reduce the pain^46^, ^47^ and the loss of appetite as well as improve fatigue symptoms of cachectic cancer patients^48^ and the overall QoL.^49^ The majority of cancer patients at advanced stages commonly suffer from reduced appetite, fatigue, pain, and weakness, which constituted an identifiable symptom cluster for cancer anorexia‐cachexia.^50^ Besides the physical symptoms, cancer‐related fatigue is also related to cognitive difficulties, insomnia and mental health problems such as anxiety and depression and reduced QoL.^51^ In addition, undergoing chemotherapy may aggravate those existing symptoms of cancer patients.^49^, ^52^ Findings from the previous studies demonstrated that individualized prescribed diets, which were made appropriate and sufficient to meet the patients’ demanded intake, could potentially empower the patient with a sense of control and be an exceedingly effective approach to psychological modulation and performance status improvement.^53^, ^54^

In addition to being malnutrition, having low prealbumin was also associated with worse QoL. Prealbumin is a visceral liver‐synthesized protein and has been used as a sensitive indicator for defining malnourishment patients with cancer.^55^, ^56^ The biological half‐life of prealbumin is approximately 2.5 days, which is not altered by stress or acute inflammation.^57^ Therefore, prealbumin is a reliable marker, and a higher serum level is related to a better prognosis, decreased complications of treatment, and increased patients’ QoL.^58^, ^59^ The result also revealed that patients from urban areas were less likely to have a higher QoL. This was similar to a previous study assessing the QoL of patients from rural and urban areas, which showed that those living in big cities and metropolises had more considerable anxiety about the deterioration of their well‐being, compared to those in rural communities.^60^

Our study suggests that an early individualized nutritional intervention during chemotherapy is feasible to improve the dietary intake as well as the QoL, especially among those prone to suffer from malnutrition and to report the worse QoL. High‐protein intake should be recommended in order to enhance muscle mass and strengths, which positively affect the physical and role function and increase the overall QoL of cancer patients. However, it should be noticed to consider the protein intake of cancer patients with chronic kidney diseases based on each individual's case. Early and intensive nutritional support also plays an integral part in minimizing the physical symptoms of cancer patients who generally undergo chemotherapy.

The strength of our study is that we used international scales such as PG‐SGA and EORTC, to assess nutritional status and QoL of cancer patients, which increased the generalizability of the findings. However, several limitations should be noticed. Our study did not cover the potential health risk behavior of patients, such as alcohol use or smoking that could act as the covariates affecting the models’ results. In addition, the study design was quasi‐experiment and the results were evaluated in a relatively short period of time, 2 months after the intervention. Thus, future nutritional intervention studies could utilize the Randomized Controlled Trials study design in a large sample size to increase the reliability of the findings. In fact, researchers built a sample menu with a calculation of the diet energy and the amount of protein intake according to the Vietnamese Food Composition Table. We continued quantifying the sample menu (quantitative energy test in the laboratory). After that the patient consumed the meal to calculate the percentage of food consumption, combined with using formula milk to adjust dietary energy as well as the amount of protein intake. The patient continued using same diet at the next time. However, it should be noticed that in the next follow‐up times, the research team consulted and monitored two times/week for the intervention group on how to adjust the diet to achieve the goal, without analysis of the energy and nutrients composition of each meal on each day. Patients who did not completely consume their meal would be instructed to increase the amount of supplemental milk per day (usually 1–3 standard cups/day) depending on their needs and dietary intake.

## 5 CONCLUSION

In conclusion, nutritional therapy with high‐protein and energy intake was beneficial to the improvement in QoL and physical function as well as the reduction of negative symptoms among gastrointestinal cancer patients. Early individualized nutritional support in consultation with professional dietitians during chemotherapy plays an integral part in enhancing the QoL and better treatment prognosis of the patients.

## CONFLICT OF INTEREST

The authors reported no conflicts of interest.

## ETHICAL STATEMENT

Written informed consent was provided to patients prior to the research. Participants were able to refuse or withdraw from the study at any time, which did not disrupt their treatment process. Ethical of the study was approved by the ethics committee of the Hanoi Medical University, Vietnam (no. 187/HĐĐĐĐHYHN).

## Supporting information

Supplementary Material

## Data Availability

The data that support the findings of this study are available on request from the corresponding author. The data are not publicly available due to privacy or ethical restrictions.
