# Supplementary material for: Nutrition intervention is beneficial to the quality of life of patients with gastrointestinal cancer undergoing chemotherapy in Vietnam
Source: Cancer Med. 2021 Feb 7;10(5):1668–80. doi: 10.1002/cam4.3766 (PMC7940238; doi:10.1002/cam4.3766)
Supplement: Supplementary file 1 — Supplementary Material [file CAM4-10-1668-s001.docx]

**Appendix S1**

**Sample menu: Energy 1900-2000Kcal/day**

| **Food** | **Menu** | **Unit of measurement** |
| --- | --- | --- |
| **Breakfast** | **Beef noodle (01 large bowl)** | |
|  | Beef noodle  Flat noodle 150g  Beef 40g  Bone broth  Mung bean sprouts 50g | 1 large bowl  7-8 small pieces |
| **Snack** | **Leanmax Hope** 54g | 6 teaspoon + 180 ml warm water |
| **Lunch** | **Rice, Stew pork, tofu braised with tomato** | |
|  | Rice 70g  Pork 50g  Tofu 50g  Cooking oil 5ml  Tomato 20g  Chayote 150g  Banana 100g | 1 small bowl  4-6 average pieces  1 large piece  1 teaspoon 5ml  1 small tomato  1 small bowl |
| **Snack** | **Shrimp pumpkin soup (100ml)** | |
|  | Sea-shrimp 30g  Pumpkin 30g  Tapioca 10g  Vegetable oil 5ml | 2-3 shrimps  1 teaspoon |
| **Dinner** | **Rice, ground meat, fried-crap, stir-cabbage** | |
|  | Rice 70g  Ground meat 30g  Carp 70g  Cooking oil 10ml  Stir-cabbage 200g  Orange 100g | 1 small bowl  2 teaspoon  1 full small bowl  1 orange without skin |
| **Snack** | **Leanmax Hope** 54g | 6 teaspoon + 180 ml warm water |

| *** 100g rice is equivalent to**  - 100g clear-noodle, rice noodle  - 250g flat noodle  - 300g fresh rice noodle  - 300g potatoes | ***100g pork is equivalent to**  - 100g beef, chicken  - 120g fresh shrimp  - 40g salted shredded pork  - 2 duck-eggs  - 3 chicken-egg  - 2 large pieces of tofu |
| --- | --- |

**Appendix S2**

**NUTRITIONAL SUPPLEMENTS FOR SNACKS**

Lean Max Hope formula milk

Ingredients: Vegetable fats, Whey protein, Soy Protein, Maltodextrin, Milk protein, Essential amino acids (Arginine, Glutamic acid, Leucine, Isoleucine, Valine), FOS (fructooligosaccharide)/Inulin, Minerals (Na, K, Cl, Ca, P, Mg, Fe, Mn, Zn, Cu, I, Se, Cr, Mo), Omega 3, Omega 6, MCT (Medium Chain Triglycerides), Synthetic Vanilla Flavor, Vitamin (A, D3, E, K, C, B1, B2, B6, B12), Niacin, Folic acid, Pantothenic acid, Biotin, Nucleotides, Curcumin. Net weight: 40g ± 10%, 400g ± 10%, 900g ± 10%, 1.8kg ± 10%.

Main quality criteria: Unit ̣ (In 100g of powder)

**Composition of nutrients in supplements**

| Energy | Kcal | 506 |
| --- | --- | --- |
| Protein | g | 20 |
| Leuxin | g | 2.9 |
| Arginin | g | 1.4 |
| Glutamic | g | 6.3 |
| Fats | g | 26 |
| MUFA | g | 4.1 |
| PUFA | g | 1.3 |
| Omega 3 | mg | 30 |
| Omega 6 | mg | 120 |
| Glucose | g | 48 |
| FOS | g | 3.5 |
| Curcumin | mg | 80 |
| Nucleotieds | mg | 25 |
| Vitamin A | mcg | 550.0 |
| Vitamin D3 | mcg | 12.0 |
| Vitamin E | mg | 11.8 |
| Vitamin K | mcg | 33.0 |
| Vitamin C | mg | 126.0 |
| Vitamin B1 | mg | 1.0 |
| Vitamin B2 | mg | 0.9 |
| Niacin (Vitamin B3) | mg | 16.8 |
| Axit Pantothenic (Vitamin B5) | mg | 5.2 |
| Vitamin B6 (Pyridosin) | mg | 1.0 |
| Folic acid B9 | mcg | 112.0 |
| Vitamin B12 (Cobalamin | mcg | 7.1 |
| Biotin | mcg | 25.2 |
| Na (Sodium) | mg | 287 |
| Kali (chloride) | mg | 450 |
| Clo (Chloride) | mg | 350 |
| Canxi (Calcium) | mg | 450 |
| Photpho (Phosphorus) | mg | 350 |
| Mg (Magnesium) | mg | 84.4 |
| Fe (Iron) | mg | 3.8 |
| Zn (Zinc) | mg | 11.2 |
| Mn (Manganege) | mg | 1.4 |
| Cu (Copper) | mcg | 260.0 |
| Iod (Iodine) | mcg | 65.0 |
| Selen (Selenium) | mcg | 31.2 |
| Crom (Chromium) | mcg | 28.6 |
| Molypden (Molypdenum) | mcg | 31.2 |

**Appendix S3**

**GUIDELINES FOR PROCESSING HIGH-ENERGY SOUP FROM COMMON FOODS**

- **METHOD OF PROCESSING SOUP OF BEEF AND PUMPKINS**
- **Ingredients:**

Beef: 35g

Pumpkin: 40g

1 small onion

Butter: 1 teaspoon (or 5ml cooking oil)

Coriander: 1 teaspoon

Fish sauce, salt

- **Processing:**

Minced beef, pumpkin

Add butter/oil into heat pan, stir with the chopped onions

Add beef and pumpkins and stir

Pour the bone broth close to the surface and cover under medium heat until the pumpkin is become soft

Use the blender and add more water until the ingredients blended

Add fish sauce/salt and boil again. Before putting the pot down, add chopped coriander, stir well.


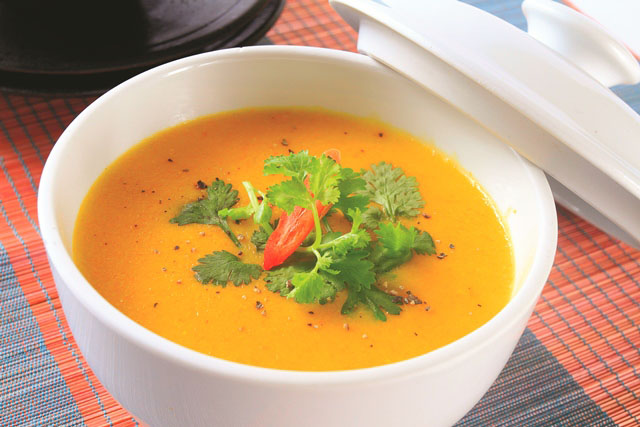


- **METHOD OF PROCESSING SOUP OF BEEF AND POTATOS**
- **Ingredients:**

Beef: 35g

Potato: 30g

Carot: 20g

1 small onion

1 teaspoon cooking oil

Tapioca flour 7g

Fish sauce, salt

- **Processing:**

Peel carrots and potatoes, onions.

[
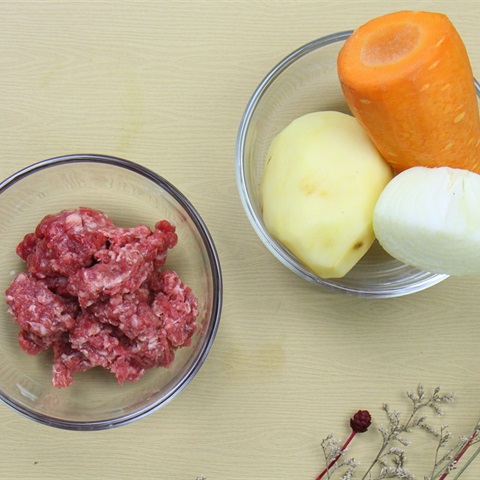
](javascript:void(0))

#### Step 1. Cut the potatoes, steam and then grind.

[
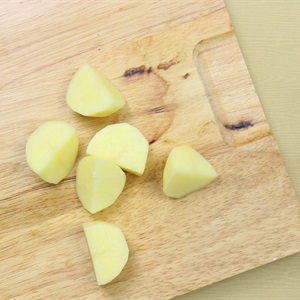

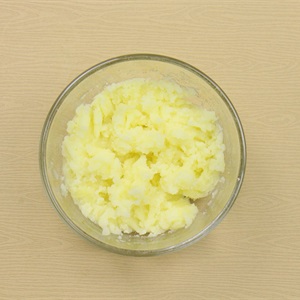
](javascript:void(0))

#### Step 2. Cut onions and carots into small pieces

[
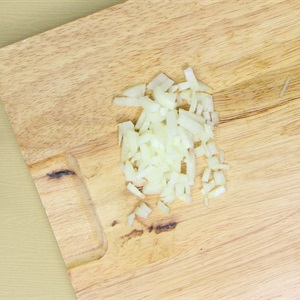

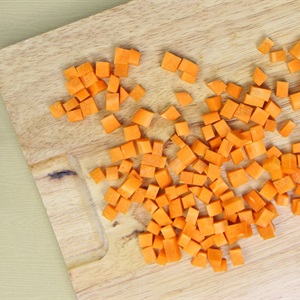
](javascript:void(0))

#### Step 3. Fry onion with 2 teaspoons of cooking oil and stir fry beef.

[
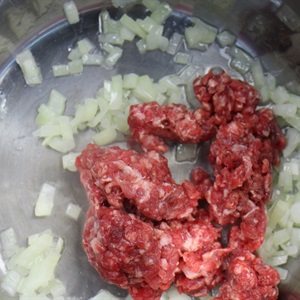

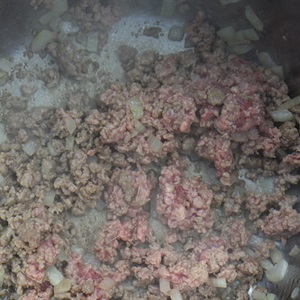
](javascript:void(0))

#### Step 4. Add carots

[
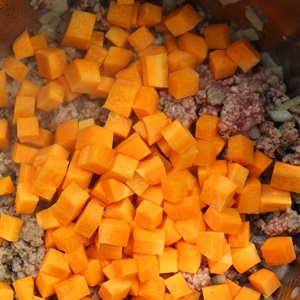

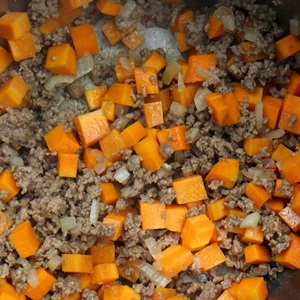
](javascript:void(0))

#### Step 5. Pour 400ml of water into the pot, cook until the carrots are soft (skim the foam that emerges during boiling water).

[
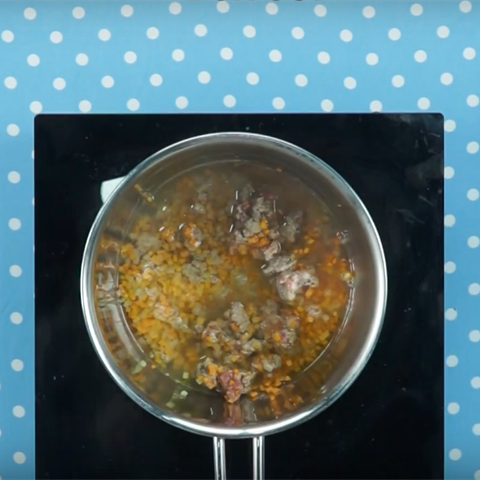
](javascript:void(0))

#### Step 6. Add steamed potatoes, stir well. Add a little tapioca flour into the soup. Turn off the heat, add salt.

[
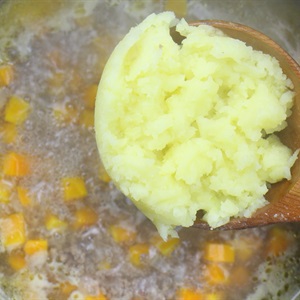

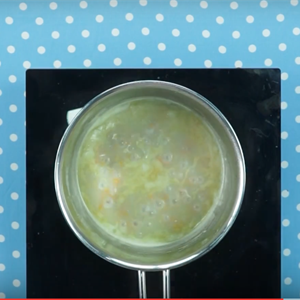
](javascript:void(0))

#### Step 7. Pour the soup into a bowl, add chopped cilantro and some ground pepper.

[
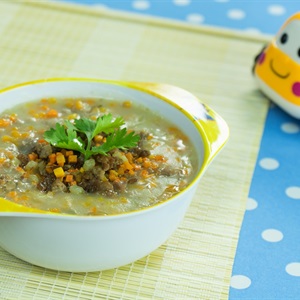

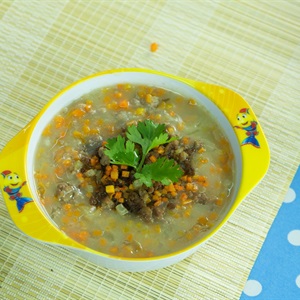
](javascript:void(0))

- **METHOD OF PROCESSING SOUP OF SHRIMP AND PUMPKINS**
- **Ingredients:**

Shrimp 40gram

Pumpkins 40gram

Rice 1 nắm

Onion, Coriander

Salt

- **Processing:**
- Step 1. Cleaning rice and then soak in water for about 2 hours for blooming, cooking quickly.


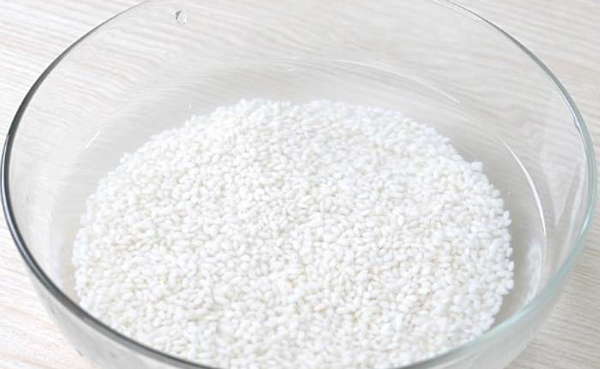


- Step 2: Peel the pumpkin and wash, cut it into small pieces. Onions and coriander washed and chopped


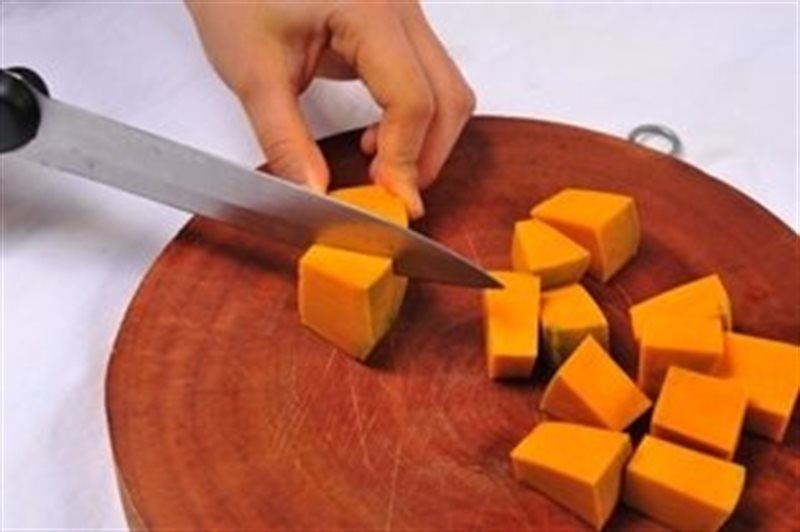


- Step 3: Cut off the head, tail, peeled, remove the black thread on the back of shrimp and then wash. Mince shrimp, marinate with salt and chopped onions.


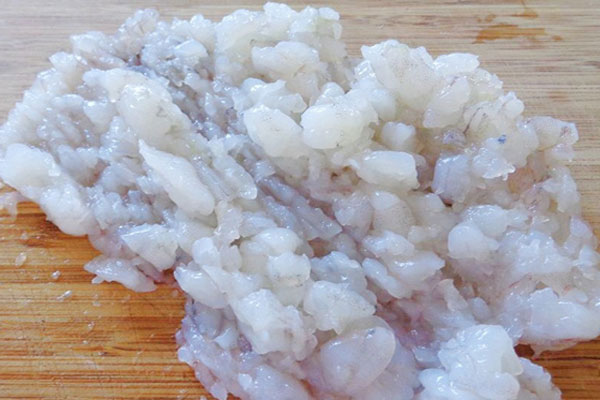


- Step 4: Pour water into the pot and boil, add rice and pumpkin at the same time and then boil. Stir well to porridge become smoothly. Continuosly add shrimp, until the shrimp is cooked, add salt and chopped scallions.
